# Reduced physical functional performance before hospitalization predicts life support limitations and mortality in nonsurgical intensive care unit patients

**DOI:** 10.5935/0103-507X.20220011-en

**Published:** 2022

**Authors:** Jamile Caroline Garbuglio Araujo da Silva, Tiago Giraldi, Carolina Matida Gontijo Coutinho, Marco Antonio Carvalho Filho, Dario Cecílio Fernandes, Thiago Martins Santos

**Affiliations:** 1 Faculdade de Ciências Médicas, Universidade Estadual de Campinas - Campinas (SP), Brazil.; 2 University Medical Center Groningen - Holland, Groningen, Netherlands.

**Keywords:** Physical functional performance, Karnofsky Performance Status, Activities of daily living, Palliative care, Intensive care units

## Abstract

**Objective:**

To assess whether scales of physical functional performance and the surprise question (“Would I be surprised if this patient died in 6 months?”) predict life support limitations and mortality in critically ill nonsurgical patients.

**Methods:**

We included 114 patients admitted from the Emergency Department to an intensive care unit in this prospective cohort. Physical functional performance was assessed by the Palliative Prognostic Score, Karnofsky Performance Status, and the Katz Activities of Daily Living scale. Two intensivists responded to the surprise question.

**Results:**

The proposed physical functional performance scores were significantly lower in patients with life support limitations and those who died during the hospital stay. A negative response to the surprise question was more frequent in the same subset of patients. Adjusted univariable analysis showed an increased odds ratio for life support limitations and death regarding the activities of daily living scale (1.35 [1.01 - 1.78] and 1.34 [1.0 - 1.79], respectively) and a negative response for the surprise question (42.35 [11.62 - 154.43] and 47.79 [11.41 - 200.25], respectively); with a p < 0.05 for all results.

**Conclusion:**

All physical functional performance scales showed lower scores in nonsurvivors and patients with life support limitations. The activities of daily living score and the surprise question increased the odds of life support limitations and mortality in our cohort of nonsurgical intensive care unit patients admitted from the Emergency Department.

## INTRODUCTION

In recent decades, advances in the management of critically ill patients have greatly contributed to a decrease in mortality in the intensive care unit (ICU).^(1)^ The maintenance of full medical treatment (FMT) is relatively straightforward for previously healthy patients with a treatable or curable disease and who have a reasonable chance of survival. However, the increasing prevalence of chronic diseases, coupled with an increase in the age of the population, might significantly worsen the prognosis of acute critical diseases. Patients with a low likelihood of surviving with an acceptable quality of life may be eligible for withholding and/or withdrawing futile therapeutic measures. Thus, the estimation of outcomes, in addition to being necessary, is an important challenge for intensive care physicians.

Scoring systems have been widely used to predict the chance of death in the ICU. For example, the Simplified Acute Physiology Score 3 (SAPS 3) and the Acute Physiology and Chronic Health Evaluation II (APACHE II) use data from physiologic parameters, admission diagnosis, and chronic health conditions to predict ICU mortality. Originally developed for patients with sepsis, the Sequential Organ Failure Assessment (SOFA) assesses disease severity during ICU stay and has also been used as a predictor of mortality.^(2)^ Comorbid conditions are also often associated with clinically relevant results. The Charlson Comorbidity Index (CCI) has been the most extensively studied and most widely used comorbidity index in medical literature when mortality is the outcome of interest.^(3)^ These scoring systems have some disadvantages, such as a large number of input variables (which might lead to missing data), lack of calibration to different ICU populations, and overestimation of the risk of death.^(4,5)^ They are also not designed to predict life support limitations (LSLs) and may not achieve precise clinical decisions in individualized patients.^(6)^

There are several physical functional performance (PFP) scales that are useful to predict outcomes in clinical contexts other than the ICU.^(7)^ The Karnofsky Performance Status (KPS) scale classifies patients according to the degree of their functional disabilities and loss of autonomy. The KPS scale is widely used in oncology to determine the ability of a patient to tolerate chemotherapy. The Katz Activities of Daily Living (ADL) scale was originally designed to evaluate performance in self-care activities in elderly patients.^(8,9)^ The Palliative Prognostic Score (PaP) was originally developed to predict the 30-day mortality of patients with solid tumors referred to palliative care centers.^(10)^ Moreover, clinical intuition may improve the precision of objective prognostic models utilizing the surprise question. The attending physicians were asked if they would be surprised if the patient died within the next 6 months. A negative response (“no, I would not be surprised”) to the surprise question predicts 6-month mortality, combined with objective scores.^(11,12)^

In this study, we hypothesized that the scales used in other clinical situations, namely, the KPS, the ADL, the PaP score, and a negative response to surprise question, would be able to predict LSL and mortality in critically ill patients admitted to a medical ICU. Our objective was to assess whether scales of physical functional performance and the surprise question (“Would I be surprised if this patient died in 6 months?”) predict life support limitations and mortality in critically ill nonsurgical patients.

## METHODS

This was a prospective, observational cohort study carried out from August 2018 to February 2019 in an adult ICU of a tertiary public university hospital in the city of Campinas (SP), Brazil. The ICU is composed of ten beds dedicated to the care of nonsurgical patients admitted from the Emergency Department. The nursing technician-to-patient ratio was 2:1, the nurse-to-patient ratio was 5:1, and the doctor-to-patient ratio was 10:1. All adult patients over 18 admitted to the ICU during the study period were eligible for inclusion after they or their relatives signed the informed consent form. A family member or guardian signed the informed consent form when the patient was not in clinical condition to do it independently. Exclusion criteria included nonconsent to participate in the study, pregnant women (as they are referred to another facility), and patients with potential follow-up difficulties, such as indigenous people and prisoners.

The research Ethics Committee of the *Universidade Estadual de Campinas*, according to CAAE 87042318.8.0000.5404, approved this prospective cohort study.

The data were collected from medical records and transferred to a form designed exclusively for this research (Appendix 1). The main author conducted the interviews with patients (when possible) and/or their families or legal representatives for information on functional capacity before hospitalization.

The patient’s functional status before hospitalization was measured using scales that assess PFP, such as the KPS and ADL. The KPS is a score ranging from zero to 100% in which every ten point drop is related to a specific autonomy and performance loss, and ADL consists of six questions, including information about bathing, dressing, toileting, transferring, eliminating, and feeding. A score closer to six indicates better function.^(8,9)^

A score including measures of the physical condition of patients in palliative care (PaP) was calculated. This score predicts 30-day survival based on the KPS and five other criteria, such as dyspnea, anorexia, clinical prediction of survival in weeks, total white blood count (WBC), and lymphocyte percentage. It ranges from zero to 17.5, with higher scores predicting a lower chance of survival.

The main author also consulted two intensivists responsible for the daily routine, who provided additional information, such as the decision of LSL during the stay in the ICU and the response to surprise questions. They did not have access to the scores, and clinical and ethical decisions were based on their clinical experiences, as well as discussions in multidisciplinary teams and daily rounds with students, residents, and ICU medical teachers. The most complex cases were referred for discussion at the weekly clinical bioethics meeting, which brought together the ICU multiprofessional team, undergraduate students, resident doctors, bioethics department professors, and chaplains. At this meeting, the technical aspects (referring to the patient’s medical management), ethical aspects (patient autonomy, beneficence, nonmaleficence), and affective aspects (the patient’s relationship with their illness, with their family and friends) were reviewed. The team deliberated and decided the course of the therapeutic plan, which could be revised if the patient presented relevant changes in their clinical status.

Continuous variables are expressed as means and standard deviations or medians and percentiles, as appropriate. Mann-Whitney test and Kruskal-Wallis test were used to assess the scales between categorical variables. A univariable analysis was conducted for the four individual scales and two dependent variables: death during hospitalization, and limitations of life support. Subsequently, the models were adjusted by SAPS3 score, CCI score, and age. The level of significance for the study was 5%. For statistical analysis, the Statistical Analysis System (SAS) for Windows, version 9.4 (SAS Institute Inc, 2002-2008, Cary, NC, USA) was used. Sample size calculation was performed using Raosoft® online calculator (http://www.raosoft.com/samplesize.html) with 95% confidence level, a margin of error of 7%, a population size of 230 (average of patients admitted to our ICU in the 6 months prior to the beginning of our study), and a 50% response distribution. The sample size was estimated at 107 subjects.

## RESULTS

The clinical characteristics at admission are shown in table 1. The majority of the 114 patients included were male (59.6%). The average age of the studied population was 57.6 years old, with more than 50% of patients over 60 years of age. Systemic arterial hypertension was the most common comorbidity (46.5%), and sepsis was the most common cause of admission to the ICU (44.7%). During ICU admission, the majority of patients were mechanically ventilated. The ICU mortality was 32.5%. Considering the deaths that occurred in the ICU and those that occurred in the ward, the total mortality was 50.9%.

**Table 1:** Patient characteristics and differences between survivors and nonsurvivors

Variables	Survivors (n = 56)	Nonsurvivors (n = 58)	Total (N = 114)	p value
Age	52.80 ± 16.93	62.26 ± 14.24	57.6 ± 16.3	< 0.0001
Sex male	43 (76.8)	25 (43.1)	68 (59.7)	
IMV	40 (71.4)	53 (91.4)	93 (81.6)	
ICU in days	14.1 ± 17.3	13.5 ± 11.8	14.0 ± 15.6	
Reason for admission				
Sepsis	30 (53.6)	21 (36.2)	51 (44.7)	
Acute respiratory failure	6 (10.7)	5 (8.6)	11 (9.6)	
AKI	4 (7.1)	5 (8.6)	9 (7.9)	
Cardiac arrest	1 (1.8)	6 (10.3)	7 (6.1)	
Acute cerebrovascular disease	3 (5.3)	3 (5.2)	6 (5.3)	
Other diseases	12 (21.4)	18 (31.0)	30 (26.4)	
Comorbidities				
Hypertension	23 (41.1)	30 (51.7)	53 (46.5)	
Diabetes	17 (30.3)	21 (36.2)	38 (33.3)	
Smoking	17 (30.3)	21 (36.2)	38 (33.3)	
Alcoholism	10 (17.8)	16 (27.6)	26 (22.8)	
Chronic kidney disease	3 (5.3)	10 (17.2)	13 (11.4)	
Congestive heart failure	5 (8.9)	7 (12.1)	12 (10.5)	
Drug abuse	6 (10.7)	4 (6.9)	10 (8.8)	
Cirrhosis	3 (5.3)	5 (8.6)	8 (7.0)	
Acute myocardial infarction	6 (10.7)	1 (1.7)	7 (6.1)	
Cerebrovascular diseases	3 (5.3)	3 (5.2)	6 (5.3)	
COPD	2 (3.6)	3 (5.2)	5 (4.4)	
Cancer	2 (3.6)	2 (3.4)	4 (3.5)	
Prognostic scores				
APACHE II	17.25 ± 7.92	24.97 ± 7.14	21.2 ± 8.4	< 0.001
SAPS 3	58.14 ± 13.32	72.93 ± 14.63	65.7 ± 15.8	< 0.001
SOFA	6.45 ± 3.77	9.50 ± 3.71	8.0 ± 4.0	0.0001
Charlson	2.79 ± 2.62	4.33 ± 2.51	3.6 ± 2.7	0.0007
Performance scales				
PaP	2.5 ± 1.5	3 ± 3.6	2.5 ± 2.9	0.0054
KPS	70 ± 20.9	60 ± 23.0	60 ± 22.5	0.0242
ADL	6 ± 1.0	6 ± 1.7	6.0 ± 1.4	0.0041
Negative response to SQ	13 (23.2)	44 (75.8)	57 (50.0)	

IMV - invasive mechanical ventilation; ICU - intensive care unit; AKI - acute kidney injury; COPD - chronic obstructive pulmonary disease; APACHE II- Acute Physiology and Chronic Health Evaluation II; SAPS 3 - Simplified Acute Physiology Score 3; SOFA -Sequential Organ Failure Assessment; PaP - Palliative Prognostic Score; KPS - Karnofsky Performance Status; ADL - Index of Independence in Activities of Daily Living; SQ - surprise question. Results expressed as median ± standard deviation or n (%).

Table 1 also shows the differences between survivors and nonsurvivors in terms of clinical characteristics, PSP scores, and the surprise question. Deceased patients were older and had worse PSP scores, as well as a more frequent negative response to the surprise question. The same occurred in patients who had LSL (Table 2).

**Table 2:** Patient characteristics and differences between full medical treatment and life support limitation

Variables	FMT (n = 71)	LSL (n= 43)	Total (N = 114)	p value
Age	55.93 ± 17.10	60.40 ± 14.51	57.6 ± 16.3	0.0106
Sex male	41 (57.7)	27 (62.8)	68 (59.7)	
IMV	54 (76.0)	39 (90.7)	93 (81.6)	
ICU in days	13.4 ± 17.04	14.7 ± 12.6	14.0 ± 15.6	
ICU survival	58 (81.7)	19 (44.2)	77 (67.5)	
Reason for admission				
Sepsis	38 (53.5)	13 (30.2)	51 (44.7)	
Acute respiratory failure	9 (12.7)	2 (4.6)	11 (9.6)	
AKI	6 (8.4)	3 (7.0)	9 (7.9)	
Cardiac arrest	3 (4.2)	4 (9.3)	7 (6.1)	
Acute cerebrovascular disease	3 (4.2)	3 (7.0)	6 (5.3)	
Other diseases	12 (10.6)	18 (41.9)	30 (26.4)	
Comorbidities				
Hypertension	31 (27.2)	22 (51.2)	53 (46.5)	
Diabetes	23 (20.2)	15 (34.9)	38 (33.3)	
Smoking	25 (21.9)	13 (30.2)	38 (33.3)	
Alcoholism	15 (13.1)	11 (25.6)	26 (22.8)	
Chronic kidney disease	6 (16.9)	7 (16.3)	13 (11.4)	
Congestive heart failure	6 (16.9)	6 (14.0)	12 (10.5)	
Drug abuse	7 (9.8)	3 (7.0)	10 (8.8)	
Cirrhosis	5 (7.0)	3 (7.0)	8 (7.0)	
Acute myocardial infarction	5 (7.0)	2 (4.7)	7 (6.1)	
Cerebrovascular diseases	3 (4.2)	3 (7.0)	6 (5.3)	
COPD	3 (4.2)	2 (4.7)	5 (4.4)	
Cancer	2 (2.8)	2 (4.7)	4 (3.5)	
Prognostic scores				
APACHE II	19.31 ± 8.31	24.26 ± 7.81	21.2 ± 8.4	0.0054
SAPS 3	62.32 ± 15.29	71.19 ± 15.22	65.7 ± 15.8	0.0057
SOFA	7.51 ± 4.34	8.81 ± 3.35	8.0 ± 4.0	0.0971
Charlson	3.11 ± 2.46	4.33 ± 2.84	3.6 ± 2.7	0.0368
Performance scales				
PaP	2.5 ± 2.2	3.5 ± 3.6	2.5 ± 2.9	0.0018
KPS	80 ± 21.2	60 ± 23.4	60 ± 22.5	0.0192
ADL	6 ± 1.1	6 ± 1.7	6.0 ± 1.4	0.0115
Negative response to SQ	17 (23.9)	40 (93.0)	57 (50.0)	

FMT - full medical treatment; LSL - life support limitation; IMV- invasive mechanical ventilation; ICU - intensive care unit, AKI -acute kidney injury; COPD - chronic obstructive pulmonary disease; APACHE II - Acute Physiology and Chronic Health Evaluation II; SAPS 3 - Simplified Acute Physiology Score 3; SOFA - Sequential Organ Failure Assessment; PaP Palliative Prognostic Score; KPS -Karnofsky Performance Status; ADL - Index of Independence in Activities of Daily Living; SQ - surprise question. Results expressed as median ± standard deviation or n (%).

Although FMT was the most frequent decision, LSL was decided in approximately one-third of cases. The most common reason for the LSL decision was the lack of clinical response to aggressive therapy, which occurred in 12 patients. Other reasons included chronic diseases (11 patients), multiorgan failure (eight patients), and poor neurologic outcomes (seven patients). The average ICU stay was 14 days and greatly varied between patients. Seventeen patients from our cohort were discussed at the bioethics meeting.

The patients on LSL had worse scores on the PaP, KPS, and ADL scales than those who received FMT. The deceased patients had worse scores on the scales and were older. The characteristics of the patients according to the outcome are shown in table 2.

## Univariable and multivariable analyses

Table 3 shows univariable and multivariable results for the KPS, PaP, ADL, and the surprise question regarding LSL. In this first crude assessment, all scores analyzed were associated with LSL. In the multivariable analysis adjusted for SAPS3 score, CCI score, and age, the ADL score and the surprise question remained significantly associated with LSL.

**Table 3:** Life support limitations

	Crude analysis*		Adjusted analysis^†^	
	OR (95%CI)	p value	OR (95%CI)	p value
KPS	1.02 (1.0 - 1.04)	0.01	1.02 (1.0 - 1.04)	0.08
PaP	0.98 (0.95 - 1.01)	0.3	0.97 (0.95 - 1.02)	0.45
ADL	1.35 (1.01 - 1.78)	0.04	1.34 (1.0 - 1.79)	0.05
SQ	42.35 (11.62 - 154.43)	0.00	47.79 (11.41 - 200.25)	0.00

OR - odds ratio; 95%CI - 95% of confidence interval; KPS - Karnofsky Performance Status; PaP - Palliative Prognostic Score; ADL - Index of Independence in Activities of Daily Living; SQ - surprise question.

* Univariate analysis; †multivariable analysis adjusted for the Simplified Acute Physiology Score 3 score, Charlson score, and age.

The same assessment was performed to assess the odds ratio (OR) of the three studied PFP scales and the surprise question for death. Additionally, for this outcome, the ADL scale and the surprise question were associated with death during hospitalization (Table 4).

**Table 4:** Hospital mortality

	Crude analysis*		Adjusted analysis*	
	OR (95%CI)	p value	OR (95%CI)	p value
KPS	1.02 (1.0 - 1.04)	0.01	1.02 (0.99 - 1.04)	0.15
PaP	0.94 (0.86 - 1.02)	0.13	0.94 (0.86 - 1.03)	0.17
ADL	1.49 (1.04 - 2.09)	0.03	1.58 (1.10 - 2.26)	0.01
SQ	10.4 (4.38 - 24.66)	0.00	6.17 (2.43 - 15.64)	0.00

OR - odds ratio; 95%CI - 95% of confidence interval; KPS - Karnofsky Performance Status; PaP - Palliative Prognostic Score; ADL - Index of Independence in Activities of Daily Living; SQ - surprise question.

* Univariable analysis.

## DISCUSSION

In our analysis, we aimed to use scales that would reproduce a more detailed anamnesis regarding the degree of functional capacity loss reported. Thus, we sought to include data from the patient’s previous functional history (KPS, ADL, and PaP), chronic diseases (CCI), physiological laboratory data (SAPS 3, APACHE II, and SOFA), and the subjective analysis of the attending physician about the prognosis through the surprise question. Although both diseased patients and those with LSL had significantly worse PFP scores, our adjusted analysis found that ADLs and a negative response to the surprise question were related to increased odds of both outcomes.

A seminal European study including thirty-seven ICUs in 17 countries showed that the most frequent reasons for the limitation of curative therapies were the lack of response to FMT, severe neurological damage, chronic diseases limiting life quality and expectancy, and multiorgan failure. Thus, despite the small number of patients in our cohort, we observed the same pattern.^(13)^

A multicenter study with adults over 80 years of age admitted to European ICUs showed that almost one-third of the 3,920 patients included had an important prior functional decline, assessed by the ADL scale of less than four. This subset of patients had significantly fewer chances of surviving after a month of hospitalization.^(14)^ In our study, most patients had an ADL score above four, and even so, mortality was significantly higher when there was a loss of independence for any of the components of this scale. In this regard, a study with 223 patients observed that the functional decline assessed by ADL lower than three was related to a fourfold likelihood of having a nosocomial infection and that half of these infections occurred within 12 days of hospitalization.^(15)^ As nosocomial infections increase hospital mortality, it is possible to trace a cause-and-effect relationship between functional decline, susceptibility to sepsis, and worsening prognosis. This result points to the need for a detailed understanding of daily activities, from simpler to more complex ones. Since we observed that a decrease in the ADL score was a predictor of LSL and hospital mortality in both unadjusted and adjusted univariate analyses, this scale seems to be suitable not only for recognizing patients with increased odds of dying during the hospital stay but also for considering LSL, especially in situations in which the patient is not responding to the treatment.

Although the surprise question is considered a subjective assessment, several studies suggest that it can improve the accuracy of prognostic indices. A survey of patients admitted to the medical ICU of a large academic medical center showed that, when associated with prognostic models, the negative response to surprise question showed strong discrimination to predict hospital mortality.^(16)^ The same occurred when both doctors and nurses were asked and gave a negative response to the surprise question in different clinical settings, such as primary care, oncology, and hemodialysis clinics.^(17,18)^

The explanation may lie in how clinical reasoning works, which, according to dual-process theory, is composed of two systems. The first, considered intuitive, is the result of the observer’s experiences and uses the recognition of environmental and patient-related factors, which together constitute patterns that generate subconsciously processed automatic responses. The second system, called analytical, works consciously and actively. In this case, the assessment is conscious and based on stimuli and constituents of the environment and the patient. Regarding clinical reasoning, these two systems operate freely in an interdependent and complementary way.^(19)^ The first system can act as an alarm to something that is not right and that could be related to the results we obtained from the surprise question, both with LSL and an increased chance of death. The surprise question requires an immediate categorical response (yes or no), in which intensive care providers take into account their intuition, built from their previous experiences, associated with the amount of information available about the patient (such as disease history, reason of hospitalization, and current acute organ dysfunctions).^(20)^ Thus, this information reinforces the interaction of the first system (the immediate response to surprise question) with the second (more analytical, which could be related to the PFP scales).

Therefore, a better individual therapeutic plan might be reached, including the odds of LSL and death. However, despite our significant results concerning both the progression to LSL and the increased risk of death, we do not advocate the idea of using the surprise question alone for decision-making in the care of critically ill patients. It should serve as an increased risk alert for these outcomes.

Our results suggest that the surprise question, along with the ADL scale, could not only be incorporated into the collection of clinical history with patients and family members but could also serve as an inspiration for clinicians to learn more about patients’ biographies.

A strength of our study is its prospective design in a nonsurgical ICU, where most patients were of advanced age and diagnosed with sepsis, a common profile worldwide. Another strong point includes the participation of two intensivists with more than 10 years of experience who work in the ICU, one in the morning and another in the afternoon, adding a workload of 30 hours each per week.

Our study had some important limitations. First, it was performed in a single center and with a small number of patients, as half of the sample was excluded for not signing the informed consent form. Thus, the results may not apply to other institutions, and studies with larger sample sizes and longer follow-up periods are needed to be clinically useful and scientifically reliable. Second, the duration of the follow-up was short, and the long-term outcomes could not be assessed. Third, despite being the most studied measure of functional status in the literature, frailty syndrome was not evaluated in our cohort.

## CONCLUSION

The assessment of functional capacity, especially employing the activities of daily living scale and the surprise question (with a negative response), were both associated with therapeutic limitation and an increased chance of death during hospitalization. Our results may assist in the development of future prognostic and screening systems in the intensive care unit.

## References

[1] Haas JS, Teixeira C, Cabral CR, Fleig AH, Freitas AP, Treptow EC (2013). Factors influencing physical functional status in intensive care unit survivors two years after discharge. BMC Anesthesiol.

[2] Lee SH, Kim SJ, Choi YH, Lee JH, Chang JH, Ryu YJ (2018). Clinical outcomes and prognostic factors in patients directly transferred to the intensive care unit from long-term care beds in institutions and hospitals: a retrospective clinical study. BMC Geriatr.

[3] Bannay A, Chaignot C, Blotière PO, Basson M, Weill A, Ricordeau P (2016). The best use of the Charlson Comorbidity Index with electronic health care database to predict mortality. Med Care.

[4] Soares M, Dongelmans DA (2017). Por que não devemos usar o APACHE II como parâmetro para avaliação de desempenho e comparação?. Rev Bras Ter Intensiva.

[5] Keegan MT, Soares M (2016). O que todo intensivista deveria saber sobre os sistemas de escore prognóstico e mortalidade ajustada ao risco. Rev Bras Ter Intensiva.

[6] Detsky ME, Harhay MO, Bayard DF, Delman AM, Buehler AE, Kent SA (2017). Discriminative accuracy of physician and nurse predictions for survival and functional outcomes 6 months after an ICU admission. JAMA.

[7] Johnson MJ, Bland JM, Davidson PM, Newton PJ, Oxberry SG, Abernethy AP (2014). The relationship between two performance scales: New York Heart Association Classification and Karnofsky Performance Status Scale. J Pain Symptom Manage.

[8] Lino VT, Pereira SR, Camacho LA, Ribeiro Filho ST, Buksman S (2008). Adaptação transcultural da Escala de Independência em Atividades da Vida Diária (Escala de Katz). Cad Saúde Pública.

[9] Duarte YA, Andrade CL, Lebrão ML (2007). O índex de Katz na avaliação da funcionalidade dos idosos. Rev Esc Enferm USP.

[10] Scarpi E, Maltoni M, Miceli R, Mariani L, Caraceni A, Amadori D (2011). Survival prediction for terminally ill cancer patients: revision of the Palliative Prognostic Score with incorporation of delirium. Oncologist.

[11] Gulini JE, Nascimento ER, Moritz RD, Vargas MA, Matte DL, Cabral RP (2018). Predictors of death in an intensive care unit: contribution to the palliative approach. Rev Esc Enferm USP.

[12] Downar J, Goldman R, Pinto R, Englesakis M, Adhikari NK (2017). The “surprise question” for predicting death in seriously ill patients: a systematic review and meta-analysis. CMAJ.

[13] Sprung CL, Woodcock T, Sjokvist P, Ricou B, Bulow HH, Lippert A (2008). Reasons, considerations, difficulties and documentation of end-of-life decisions in European intensive care units: the ETHICUS Study. Intensive Care Med.

[14] Guidet B, de Lange DW, Boumendil A, Leaver S, Watson X, Boulanger C, Szczeklik W, Artigas A, Morandi A, Andersen F, Zafeiridis T, Jung C, Moreno R, Walther S, Oeyen S, Schefold JC, Cecconi M, Marsh B, Joannidis M, Nalapko Y, Elhadi M, Fjølner J, Flaatten H, VIP2 study group (2020). The contribution of frailty, cognition, activity of daily life and comorbidities on outcome in acutely admitted patients over 80 years in European ICUs: the VIP2 study. Intensive Care Med.

[15] Mazière S, Couturier P, Gavazzi G (2013). Impact of functional status on the onset of nosocomial infections in an acute care for elders unit. J Nutr Health Aging.

[16] Lakin JR, Robinson MG, Obermeyer Z, Powers BW, Block SD, Cunningham R (2019). Prioritizing primary care patients for a communication intervention using the “surprise question”: a prospective cohort study. J Gen Intern Med.

[17] Singh S, Graham Z, Rodriguez A, Lee D, Wenger B, Min SJ (2019). Accuracy of the surprise question on an inpatient oncology service: a multidisciplinary perspective. J Hosp Palliat Nurs.

[18] Da Silva Gane M, Braun A, Stott D, Wellsted D, Farrington K (2013). How robust is the ‘surprise question’ in predicting short-term mortality risk in haemodialysis patients?. Nephron Clin Pract.

[19] Pelaccia T, Tardif J, Triby E, Charlin B (2011). An analysis of clinical reasoning through a recent and comprehensive approach: the dual-process theory. Med Educ Online.

[20] Hadique S, Culp S, Sangani RG, Chapman KD, Khan S, Parker JE (2017). Derivation and validation of a prognostic model to predict 6-month mortality in an intensive care unit population. Ann Am Thorac Soc.

